# Point-of-Care Ultrasound for the Evaluation and Management of Posterior Cruciate Ligament Injuries: A Systematic Review

**DOI:** 10.3390/diagnostics13142352

**Published:** 2023-07-12

**Authors:** Anca Gabriela Stoianov, Jenel Marian Patrascu, Bogdan Gheorghe Hogea, Bogdan Andor, Sorin Florescu, Liviu Coriolan Misca, Ruxandra Laza, Roxana Manuela Fericean, Adelina Mavrea, Artiom Terzi, Jenel Marian Patrascu

**Affiliations:** 1 Department of Orthopedics and Traumatology, “Victor Babes” University of Medicine and Pharmacy Timisoara, 300041 Timisoara, Romania; anca.stoianov@umft.ro (A.G.S.); patrascu.jenel@umft.ro (J.M.P.); hogea.bogdan@umft.ro (B.G.H.); andor.bogdan@umft.ro (B.A.); florescu.sorin@umft.ro (S.F.); miscal.liviu@gmail.com (L.C.M.); jenel.patrascu@umft.ro (J.M.P.J.); 2 Doctoral School, “Victor Babes” University of Medicine and Pharmacy Timisoara, Eftimie Murgu Square 2, 300041 Timisoara, Romania; manuela.fericean@umft.ro; 3 Department of Infectious Diseases, “Victor Babes” University of Medicine and Pharmacy Timisoara, Eftimie Murgu Square 2, 300041 Timisoara, Romania; 4 Department of Internal Medicine I, Cardiology Clinic, “Victor Babes” University of Medicine and Pharmacy Timisoara, Eftimie Murgu Square 2, 300041 Timisoara, Romania; mavrea.adelina@umft.ro; 5 Department of General Medicine, “Nicolae Testemitanu” State University of Medicine and Pharmacy, Stefan cel Mare si Sfant Boulevard 165, 2004 Chisinau, Moldova; artiomterzimd@gmail.com

**Keywords:** point-of-care diagnostics, posterior cruciate ligament, knee injuries

## Abstract

Posterior cruciate ligament (PCL) injuries, though less common than other knee ligament injuries, pose significant management challenges. This study aimed to systematically review and analyze the current evidence on the use of point-of-care ultrasound (POCUS) for the evaluation and management of PCL injuries. It was hypothesized that POCUS has comparable diagnostic accuracy to magnetic resonance imaging (MRI) and that the use of POCUS improves patient outcomes and reduces healthcare costs. A comprehensive systematic review of articles published up to April 2023 was conducted using PubMed, Web of Science, Cochrane, and Scopus databases and adhered to the PRISMA guidelines. Studies were selected based on relevance to the research question, with a focus on diagnostic accuracy, reliability, clinical utility, and cost-effectiveness of POCUS in PCL injuries. Seven studies, analyzing a total of 242 patients with PCL injuries, were included. The reported sensitivity and specificity of POCUS for diagnosing PCL injuries ranged from 83.3% to 100% and 86.7% to 100%, respectively, across the studies. In one study, POCUS demonstrated a positive predictive value (PPV) of 87.9% and a negative predictive value (NPV) of 82.4%. Additionally, three studies reported 100% accuracy in PCL injury detection using POCUS, suggesting a substantial potential for cost savings by eliminating the need for MRI. This systematic review supports the use of POCUS in the evaluation and management of PCL injuries, suggesting that POCUS is a reliable, cost-effective tool with high diagnostic accuracy comparable to that of MRI, offering the potential to improve patient outcomes and reduce healthcare costs. The data collated in this review can inform clinical practice and guide future research in the field.

## 1. Introduction

The posterior cruciate ligament (PCL) is one of the four major ligaments of the knee, providing approximately 95% of the total restraining force to posterior tibial displacement [1]. Despite its critical role in maintaining knee stability, PCL injuries are less common than other knee ligament injuries, although they can cause significant functional impairment and early-onset osteoarthritis if not appropriately diagnosed and managed [2]. Traditional methods of assessing PCL injuries include physical examination, magnetic resonance imaging (MRI), and arthroscopy, while MRI remains the gold standard diagnostic modality [3].

Point-of-care ultrasound (POCUS) is a rapidly evolving technology and has been increasingly used in various clinical settings for its advantages such as being a non-invasive procedure, providing real-time imaging, and improving the cost-effectiveness of patient management [4]. In musculoskeletal medicine, the utilization of POCUS has expanded dramatically, enabling healthcare professionals to diagnose and manage a wide range of soft tissue injuries, including ligament and tendon injuries, in a more timely and efficient manner [5].

However, the use of POCUS in the evaluation and management of PCL injuries has not been thoroughly examined in the literature. Few studies have investigated its reliability, accuracy, and clinical applicability in diagnosing PCL injuries or providing the guidelines for treatment decisions compared to standard diagnostic tools such as MRI [6]. Moreover, there are limited data available on the potential influence of POCUS use on patient outcomes and healthcare costs in the context of PCL injuries [7].

Given the importance of early and accurate diagnosis of PCL injuries in preventing long-term knee instability and its associated complications, alternative diagnostic methods such as POCUS warrant a thorough investigation [8]. Recent advancements in ultrasound technology and improvements in operator proficiency have further highlighted the potential of POCUS as a useful diagnostic tool in musculoskeletal injuries [9,10]. Therefore, an in-depth analysis of the benefits and limitations of POCUS use for PCL injuries can significantly contribute to the literature and potentially inform future practice guidelines.

This study aims to fill the gap in the literature by systematically reviewing the available evidence on the use of POCUS for the evaluation and management of PCL injuries. The study’s hypotheses are that POCUS has a comparable diagnostic accuracy to the gold standard MRI for detecting PCL injuries and that the use of POCUS in the management of PCL injuries might lead to improved patient outcomes and reduced healthcare costs. The objectives are to systematically review and analyze the current evidence on POCUS use in PCL injuries and to make recommendations for clinical practice based on the findings.

## 2. Materials and Methods

### 2.1. Review Protocol

This systematic review was carried out in April 2023 by searching four electronic databases: PubMed, Web of Science, Cochrane, and Scopus. Literature published up until April 2023 was included. The search strategy involved using medical subject headings (MeSH) keywords like “Point-of-Care Ultrasound”, “Ultrasound”, “PCL injury”, “Posterior Cruciate Ligament”, “Diagnostic accuracy”, “Musculoskeletal ultrasound”, “Knee injury”, and “Ligament injury”. The search was restricted to articles published in the English language.

**Figure 1:**
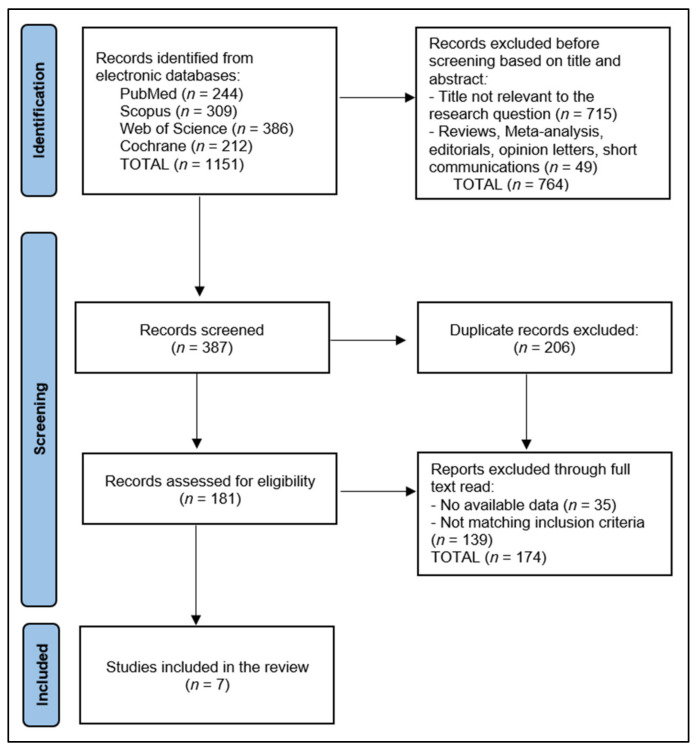
PRISMA Flow Diagram.

As described in Figure 1, the current review adhered to the Preferred Reporting Items for Systematic Reviews and Meta-Analyses (PRISMA) guidelines [11] and the International Prospective Register of Systematic Reviews (PROSPERO) criteria [12]. A structured and comprehensive search strategy was employed to identify relevant scientific papers that examine the use and effectiveness of POCUS in the diagnosis and management of PCL injuries. The systematic review was registered on the Open Science Framework (OSF) platform [13].

The review aimed to explore several research questions assessing the diagnostic accuracy, reliability, clinical utility, and cost-effectiveness of POCUS in PCL injuries, as compared with the gold standard MRI. The primary research objective was to ascertain the diagnostic accuracy of POCUS compared to standard diagnostic methods such as the MRI or surgical confirmation of PCL lesions [14].

### 2.2. Selection Process

Two independent researchers initiated the selection process by removing duplicate entries from the combined search results. They then screened titles and abstracts against our inclusion and exclusion criteria. The inclusion criteria at this stage were studies that (1) addressed the use of POCUS in evaluating and managing PCL injuries or PCL injuries in conjunction with other ligaments of the knee joint; (2) presented preliminary data in their abstracts on diagnostic accuracy, reliability, or clinical utility or made explicit references to these outcomes being measured in the full text. Exclusion criteria at this stage included (1) studies not focused on PCL injuries; (2) studies that did not mention the use of POCUS; (3) studies focused on other knee ligament injuries, not inclusive of the PCL; and (4) studies that were review articles, case reports, abstracts, commentaries, or letters to the editor.

Following the initial screening, we conducted a full-text review for the remaining articles to verify their fulfillment of the inclusion criteria. This review ensured the studies (1) provided detailed data on diagnostic accuracy, reliability, clinical utility, impact on patient outcomes, and cost-effectiveness; (2) described the POCUS technique used in a comprehensive manner; and (3) compared POCUS with the gold standard MRI or surgical confirmation of ultrasound findings. Exclusion criteria for full-text review included (1) studies not providing sufficient data on POCUS application and outcomes; (2) studies where POCUS was not described in an explicit manner; and (3) studies examining other knee ligament injuries, not inclusive of the PCL.

Following the full-text review, the reference lists of all selected articles were carefully scrutinized to identify any additional literature that may have been overlooked during the database search. In this systematic review, POCUS was compared to standard diagnostic methods such as MRI and arthroscopy, where available in the selected studies.

### 2.3. Data Extraction and Quality Assessment

The initial search generated a significant number of studies, some of which were identified as duplicates. After excluding non-relevant papers based on their abstracts, the remaining full-text articles were thoroughly examined for relevance. Finally, seven articles were selected for inclusion in the systematic review. Using the Study Quality Assessment Tools from the National Heart, Lung, and Blood Institute (NHLBI) [15], two investigators independently assessed the studies and noted their conclusions.

The variables considered for extraction from the included studies comprised first author’s name, country of publication, year of publication, study design, quality assessment score, number of patients, average age of the patients, age range or standard deviation, sex distribution, size of PCL injury, study particularities, the ultrasound device and transducer frequency that was used to assess the ligaments, accuracy of the ultrasound method measured by positive predictive value, negative predictive value, sensitivity, and specificity, and study conclusions.

The Quality Assessment Tool for Observational Cohort and Cross-Sectional Studies was used to evaluate the included articles [16]. Each question within the tool was scored with 1 for “Yes” responses and 0 for “No” and “Other” responses. Studies with scores from 0 to 4 were classified as poor quality, those with scores between 5 and 9 as fair quality, and those scoring 10 or above as excellent quality. To minimize bias and enhance reliability, two researchers independently assessed the quality of the included articles.

### 2.4. Assessment of Publication Bias

**Figure 2:**
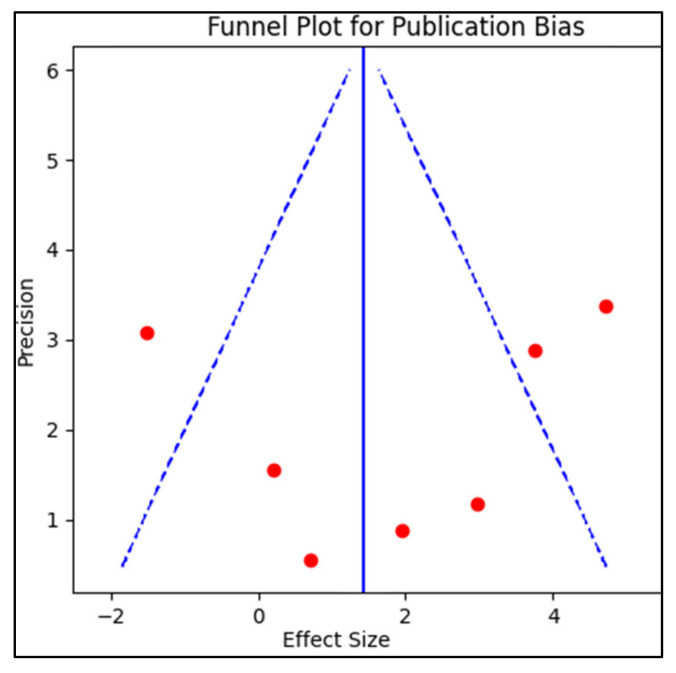
Funnel plot for publication bias.

Publication bias was examined by creating a funnel plot, where the standard error of the log odds ratio was plotted against its corresponding log odds ratio. The symmetry of the plot was visually examined and further assessed using Egger’s regression test, with a *p*-value < 0.05 indicating significant publication bias, as described in Figure 2. A sensitivity analysis was also conducted by removing one study at a time and recalculating the pooled odds ratios to evaluate the robustness of the results and to examine the impact of individual studies on the overall effect size.

## 3. Results

### 3.1. Study Characteristics

In examining the characteristics of the studies collated in Table 1, we observed a diverse geographical representation across the included research, with authors from Taiwan, Korea, India, Italy, and Japan. The studies spanned over two decades, ranging from 1991 to 2017 [17,18,19,20,21,22,23]. The study by Wang CY et al. conducted in Taiwan in 2009 was a retrospective cohort study and was appraised as having a fair study quality [17]. Similarly, the study by Lalitha P et al., carried out in India in 2010, was also a prospective cohort study and was rated as fair in terms of quality [20]. Also, a Japanese study by Suzuki S et al. from 1991, a prospective cohort study, was rated as fair [23]. However, two other studies conducted in Taiwan by Wang LY et al. in 2017 and Hsu CC et al. in 2005 were designated as a case-control study and a prospective cohort study, respectively, both of which were rated as having good quality [18,21]. Furthermore, a Korean study by Cho CH et al. from 2001 and an Italian study by Sorentino F et al. from 2008, both being prospective cohort studies, also received good quality ratings [19,22].

### 3.2. Background Characteristics

The seven studies included in the systematic review collectively analyzed 242 patients who had sustained posterior cruciate ligament (PCL) injuries [17,18,19,20,21,22,23], as described in Table 2. The average age among the patients ranged from 22 years in the study by Suzuki S et al. [23] to 42 years in the study by Cho CH et al. [19]. The sex distribution also varied across the studies, with the proportion of male patients ranging from 51.4% [19] to 89% [20]. The details of the injuries were described in different ways throughout the studies. The size of the injuries, when reported, ranged from an average of 5.6–12.0 mm in the study by Wang CY et al. [17] to 15.6 mm in the study by Cho CH et al. [19]. Hsu CC et al. [21] noted seven complete PCL ruptures, with six of them having associated anterior cruciate ligament (ACL) injuries. Sorentino F et al. [22] categorized PCL lesions as acute (73.3%) or chronic (26.7%).

Wang CY et al. [17], Cho CH et al. [19], and Suzuki S et al. [23] documented the cause of knee injuries. Falls, traffic incidents, and sports accidents were common causes. The average duration from the injury to assessment in Wang CY et al.’s study [17] was 154 days, and the average time until MRI assessment after the sonographic test was 29 days. In terms of the ultrasound examination, Cho CH et al. [19] found the echogenicity to be heterogeneously hypoechoic in 34.3% of patients, and the posterior margin of the ligament was indistinct in 31.4% of patients. Notably, Lalitha P et al. [20] found a significant correlation between the ultrasound and MRI appearance of PCL injury. Overall, these results suggest varied age distribution and etiological factors for PCL injuries and highlighted the relevance of ultrasound in evaluating these injuries, with some studies even identifying distinct sonographic patterns [19] and a strong correlation with MRI findings [20].

### 3.3. Ultrasound Assessment

Table 3 summarizes the accuracy of ultrasound assessment for the diagnosis of PCL injuries across seven studies, revealing high sensitivity and specificity percentages across the board [17,18,19,20,21,22,23]. Wang CY et al. [17] demonstrated a sensitivity of 83.3% and a specificity of 87.0% using a 7–14 MHz linear transducer. Although the positive predictive value (PPV) and negative predictive value (NPV) were not reported, the study concluded that POCUS’s effectiveness was not inferior to magnetic resonance imaging (MRI). In the study by Wang LY et al. [18], a 4–9 MHz multifrequency linear transducer was utilized, yielding a sensitivity of 90.6%, specificity of 86.7%, PPV of 87.9%, and NPV of 82.4%. This study suggested that POCUS is a reliable tool for PCL injury assessment, especially for PCL thicknesses of 6.5 mm or more.

Cho CH et al. [19] utilized a 5–10 MHz broadband linear-array transducer, demonstrating a sensitivity and specificity of 100%, indicating a perfect accuracy in detecting PCL injuries. While PPV and NPV were not reported, the study highlighted that ultrasound could be a cost-effective and precise tool for diagnosing suspected PCL injuries, potentially guiding decisions about more expensive diagnostic methods such as MRI or surgeries. Lalitha P et al. [20] reported a sensitivity of 90.9%, a specificity of 100%, a PPV of 100%, and an NPV of 99% using a 3–5 MHz transducer. These results demonstrate that ultrasound has excellent accuracy for detecting PCL injuries, with the PCL appearing as a homogeneously hypoechoic structure on ultrasound.

Hsu CC et al. [21] used a 5–10 MHz broadband linear-array ultrasound transducer and reported a sensitivity and specificity of 100%, suggesting perfect accuracy in identifying PCL lesions. The study indicated that ultrasound assessment was as accurate as the definitive diagnosis after arthroscopy. Sorentino F et al. [22] utilized a 7–12 MHz high-resolution multifrequency linear array transducer and found a sensitivity, specificity, PPV, and NPV of 100%. This study noted the high accuracy of ultrasound in detecting PCL lesions and suggested that it could be used for the follow-up of isolated chronic lesions, potentially eliminating the need for MRI and leading to substantial cost savings. Finally, Suzuki S et al. [23] used a 5.0–7.5 MHz linear and convex transducer and achieved a sensitivity, specificity, PPV, and NPV of 100%. The study concluded that ultrasound was very efficient in diagnosing PCL ruptures, especially when compared to MRI.

## 4. Discussion

### 4.1. Summary and Contributions

This systematic review aimed to assess the utility, diagnostic accuracy, and clinical value of point-of-care ultrasound (POCUS) in the evaluation and management of posterior cruciate ligament (PCL) injuries. The results from the included studies presented strong evidence for POCUS’s sensitivity and specificity for diagnosing PCL injuries. Indeed, several of the studies indicated a 100% rate for both metrics, suggesting that POCUS might be comparable, if not superior, to the more conventional diagnostic methods such as MRI and arthroscopy [17,19,21,22,23]. This corroborates prior studies emphasizing the high diagnostic accuracy of POCUS in evaluating other knee ligament injuries, such as the anterior cruciate ligament (ACL) [24].

In terms of positive and negative predictive values, the information was not uniformly reported across the studies. However, for the studies that did provide these values, POCUS demonstrated high predictive accuracy in detecting PCL injuries, as shown by previous findings [18,22,23]. Comparative studies on ACL injuries have also reported high predictive values, further emphasizing the potential of ultrasound as a diagnostic tool [25].

Another key aspect of this review was to evaluate the cost-effectiveness of POCUS and its clinical utility in managing PCL injuries. Based on our findings, ultrasound was deemed valuable in guiding clinical decision-making regarding more expensive tests, the surgical approach, or the follow-up of chronic lesions [17,19,22]. This aligns with a previous systematic review suggesting that POCUS could help reduce the need for more expensive diagnostic tools like MRI for ACL injuries [26]. Moreover, a compelling finding from our review was the diversity of frequency ranges and ultrasound devices used across studies. While this poses a challenge to standardizing the use of POCUS for PCL injuries, it also reflects the adaptability of the tool, underscoring its potential for broader application across different clinical settings. Similar versatility has been noted in the use of POCUS for ACL injuries [27].

However, the studies included in our review demonstrated varied age distribution and etiological factors for PCL injuries. Given the inconsistencies in reporting injury specifics and the varied patient demographics across studies, further research may be needed to standardize POCUS for PCL injury evaluation and management. Similar calls for standardization have been made for other knee ligament injuries, such as ACL injuries, reinforcing the need for this initiative [28].

One meta-analysis that compared ultrasound findings between ACL and PCL demonstrated that knee ultrasound was a highly effective tool for diagnosing both ligament injuries, with a pooled sensitivity of 88% and 96% specificity for ACL and 99% for both sensitivity and specificity for PCL [29]. However, the analysis found no significant difference between functional and conventional knee ultrasound in detecting ACL injuries. The traditional approach for diagnosing these injuries involved physical examination and stress radiographs, but the sensitivity and specificity of these methods were found to vary widely. For example, physical examination tests such as the anterior drawer test, Lachman test, and pivot shift test for ACL injuries demonstrated a wide range of sensitivity (38–87%) and specificity (25–100%) [30,31]. Similarly, the sensitivity of the posterior drawer test, posterior sag sign test, and quadriceps active test for diagnosing PCL injuries also varied significantly (51–98%) [32].

Stress radiography for diagnosing ACL and PCL injuries was noted to have variable sensitivity (43–100% for ACL and 88–100% for PCL) and specificity (76–100% for ACL and 77–100% for PCL), leading to challenges in establishing standardized techniques for the process [33,34]. The value of physical examinations and stress radiographs was questioned, particularly in acute phases where they may be difficult to execute accurately due to severe pain, muscle spasm, or edema. In contrast, the versatility of knee ultrasound was highlighted, offering real-time comparisons between injured and non-injured knees, as well as the potential for functional testing [35,36,37].

Other studies recommended that musculoskeletal radiologists should perform the initial ultrasound assessment on patients in a prone position to diagnose ACL and PCL injuries, as this increased the sensitivity of the test. However, if clinicians other than musculoskeletal radiologists were to perform knee ultrasounds, targeted training in musculoskeletal ultrasound would be needed to ensure diagnostic accuracy [38]. A previous meta-analysis [39] was acknowledged, but the authors noted its limitations, such as inadequate analysis of heterogeneity and use of non-hierarchical models. The present analysis only included cases with definitive diagnostic results, which could exclude patients with equivocal or inconclusive findings from ultrasound examination. Furthermore, the authors suggested future studies to investigate the cost- and time-effectiveness of ultrasound, as well as comparisons with other modalities like MRI, to solidify the position of knee ultrasound as an initial diagnostic tool in clinical practice [37,38,39,40].

In our review, we also noticed that some studies identified distinct sonographic patterns in PCL injuries and reported a strong correlation with MRI findings. This reflects POCUS’s potential to provide detailed insights into the nature of the injury, which could significantly inform the management approach [20]. Comparable findings have been noted in the sonographic evaluation of ACL tears, which exhibit characteristic patterns on ultrasound [29]. Thus, the current review presents strong evidence for the effectiveness and clinical utility of POCUS in the evaluation and management of PCL injuries. While promising, these results should be interpreted in light of the limitations of the included studies and the need for more standardized research in this field.

### 4.2. Strengths and Limitations

This systematic review, while extensive in its approach, bears several limitations that ought to be considered. The literature search was limited to articles published in English, potentially omitting relevant non-English studies and potentially introducing language bias. Moreover, substantial variability was observed across the studies in terms of the ultrasound devices and transducer frequencies utilized, which could impact the reliability and comparability of the findings. Additionally, not all studies provided complete data, including important metrics such as PPV and NPV, which could limit the strength and applicability of our conclusions. Subjective interpretations during the abstract review and quality assessment might have introduced bias. Further, although attempts were made to assess publication bias, the limited number of studies included could impede a comprehensive evaluation. One significant limitation of our systematic review is the limited geographic representation in the studies included, since, excepting one study from Italy, none of the included studies originated from the USA or other European countries. This geographic skew could potentially limit the generalizability of our findings, as the use, accessibility, and prevalence of POCUS for PCL injuries might vary across different healthcare settings and regions. Differences in medical training, healthcare infrastructure, and even patient populations might mean that our findings are less applicable to contexts outside of those represented in the studies included in our review. The reason behind this distribution is unclear, and it may simply reflect the state of the literature at the time of our search. These limitations emphasize the need for further multicenter studies with standardized methodologies to validate these findings.

## 5. Conclusions

POCUS presents as a reliable, cost-effective tool for the evaluation and management of PCL injuries. Across different geographic areas, patient demographics, and injury characteristics, ultrasound exhibited high sensitivity and specificity, comparable to the diagnostic performance of MRI and arthroscopy. These findings were consistent irrespective of the ultrasound transducer utilized, demonstrating the versatility of POCUS in a clinical setting. Notably, several studies identified distinct sonographic patterns in PCL injuries and reported a strong correlation between ultrasound and MRI findings. Furthermore, with the ability to identify PCL thicknesses of 6.5 mm or more, ultrasound may facilitate the monitoring of isolated chronic lesions. Overall, these findings underscore the substantial value of integrating POCUS into routine practice for the efficient evaluation and management of PCL injuries.

## Figures and Tables

**Table 1:** Study characteristics.

Study and Author	Country	Study Year	Study Design	Study Quality
1 [17] Wang CY et al.	Taiwan	2009	Retrospective Cohort	Fair
2 [18] Wang LY et al.	Taiwan	2017	Case-Control	Good
3 [19] Cho CH et al.	Korea	2001	Prospective Cohort	Good
4 [20] Lalitha P et al.	India	2010	Prospective Cohort	Fair
5 [21] Hsu CC et al.	Taiwan	2005	Prospective Cohort	Good
6 [22] Sorentino F et al.	Italy	2008	Prospective Cohort	Good
7 [23] Suzuki S et al.	Japan	1991	Prospective Cohort	Fair

**Table 2:** Characteristics of patients in the included studies.

Study Number	Number of Patients	Average Age (Years)	Sex (Men, %)	Size of Injury *	Particularities
1—Wang CY et al.	35	29.2 (17–55)	26 (74.3%)	5.6–12.0 mm	Knee injuries resulted from falls (9 cases), traffic incidents (14 cases), and sports accidents (12 cases). The average duration from injury to assessment was 154 days (2–1008). On average, it took 29 days (2–86) until MRI assessment after the sonographic test. Ultrasound staff had 7 years’ experience.
2—Wang LY et al.	33	38.8 (14.5)	19 (57.6%)	8.1 (3.5) mm	Joint effusions were observed in 76% of patients.
3—Cho CH et al.	35	42.0 (18–65)	18 (51.4%)	15.6 (2.5) mm	Three patients had tears in the proximal third; seven in the middle third; and five in the distal third. The echogenicity was heterogeneously hypoechoic in 34.3% patients, and the posterior margin of the ligament was indistinct in 31.4% of patients. PCL tears confirmed at surgery resulted from falls (5 cases) and traffic incidents (9 cases).
4—Lalitha P et al.	110	34.0	98 (89.0%)	NR	A significant correlation was found between the ultrasound and MRI appearance of PCL injury.
5—Hsu CC et al.	11	25.1 (7.7)	6 (54.5%)	12.0 (5.0) mm	Seven complete PCL ruptures, six of them with associated ACL injuries.
6—Sorentino F et al.	13	24.6 (20–36)	8 (61.5%)	9.2 (1.7) mm	Ten PCL lesions (73.3%) were acute, and three (26.7%) were chronic.
7—Suzuki S et al.	5	22.0 (16–37)	4 (80.0%)	NR	Knee injuries resulted from falls (1 case) and traffic incidents (4 cases).

*—Data reported as mean (SD); NR—not reported; PCL—posterior cruciate ligament; ACL—anterior cruciate ligament.

**Table 3:** Ultrasound accuracy.

Study Number	Ultrasound Device	Sensitivity (%)	Specificity (%)	PPV (%)	NPV (%)	Remarks and Conclusions
1—Wang CY et al.	7–14 MHz linear transducer 1. ATL-HDI 5000 (Advanced Technology Laboratories Inc., Bothell, WA, USA) 2. Xario SSA-660A (Toshiba Inc., Tokyo, Japan)	83.3	87.0	NR	NR	POCUS was non-inferior to MRI
2—Wang LY et al.	4–9 MHz multifrequency linear transducer S2000 US system (Siemens Healthcare, Erlangen, Germany)	90.6	86.7	87.9	82.4	POCUS is reliable for PCL injury assessment for a PCL thickness ≥ 6.5 mm. The optimal cut-off for red pixel intensity was ≤146.6.
3—Cho CH et al.	5–10 MHz broadband linear-array transducer (HDI or HDI-3000; ATL, Bothell, Wash)	100	100	NR	NR	The study indicates that ultrasound could be effective for diagnosing suspected PCL injuries, potentially guiding decisions about more expensive MRI tests or surgeries, although evidence was unclear compared with MRI, arthrotomy, or arthroscopy.
4—Lalitha P et al.	3–5 MHz transducer; Siemens Antares Ultrasound (Siemens Healthcare, Erlangen, Germany)	90.9	100	100	99	On ultrasound, PCL appears as a homogeneously hypoechoic structure. Ultrasound has a good accuracy for the detection of PCL injury.
5—Hsu CC et al.	5–10 MHz broadband linear-array ultrasound transducer (LOGIQ 700MR, General Electric Company, Milwaukee, WI)	100	100	NR	NR	Ultrasound assessment was equally accurate in identifying PCL lesions as the definitive diagnosis after arthroscopy.
6—Sorentino F et al.	7–12 MHz high-resolution multifrequency linear array transducer HDI 5000 scanner (ATL-Philips, Bothell, WA, USA)	100	100	100	100	Ultrasound had a high accuracy in detecting PCL lesions. It could be utilized for the follow-up of isolated chronic lesions, thereby eliminating the need for MRI and resulting in substantial cost savings.
7—Suzuki S et al.	5.0–7.5 MHz linear and convex transducer, Shimadzu SDU-500 (Kyoto, Japan)	100	100	100	100	The diagnosis of PCL rupture was much easier in the posterior approach. Ultrasound was very efficient in diagnosis, compared with MRI.

NR—not reported; POCUS—point-of-care ultrasound; MRI—magnetic resonance imaging; PPV—positive predictive value; NPV—negative predictive value; PCL—posterior cruciate ligament.

## Data Availability

Not applicable.
